# Surface engineered nanohybrids in plasmonic photothermal therapy for cancer: Regulatory and translational challenges

**DOI:** 10.7150/ntno.92639

**Published:** 2024-02-12

**Authors:** Monalisha Debnath, Sujit Kumar Debnath, Mangal Vishnu Talpade, Shweta Bhatt, Prem Prakash Gupta, Rohit Srivastava

**Affiliations:** Department of Biosciences and Bioengineering, Indian Institute of Technology Bombay, Mumbai, India.

**Keywords:** plasmonic, photothermal, photodynamic, NIR, nanohybrid

## Abstract

Plasmonic materials as non-invasive and selective treatment strategies are gaining increasing attention in the healthcare sector due to their remarkable optical and electronic properties, where the interface between matter and light becomes enhanced and highly localized. Some attractive applications of plasmonic materials in healthcare include drug delivery to target specific tissues or cells, hence reducing the side effects of the drug and improving their efficacy; enhancing the contrast and resolution in bioimaging; and selectively heating and destroying the cancerous cells while parting the healthy cells. Despite such advancements in photothermal therapy for cancer treatment, some limitations are still challenging. These include poor photothermal conversion efficiency, heat resistance, less accumulation in the tumor microenvironment, poor biosafety of photothermal agents, damage to the surrounding healthy tissues, post-treatment inflammatory responses, etc. Even though the clinical application of photothermal therapy is primarily restricted due to poor tissue penetration of excitation light, enzyme therapy is hindered due to less therapeutic efficacy. Several multimodal strategies, including chemotherapy, radiotherapy, photodynamic therapy, and immunotherapy were developed to circumvent these side effects associated with plasmonic photothermal agents for effective mild-temperature photothermal therapy. It can be prophesied that the nanohybrid platform could pave the way for developing cutting-edge multifunctional precise nanomedicine via an ecologically sustainable approach towards cancer therapy. In the present review, we have highlighted the significant challenges of photothermal therapy from the laboratory to the clinical setting and their struggle to get approval from the Food and Drug Administration (FDA).

## 1. Introduction

Plasmonic photothermal effects (PPE) are emerging as a fascinating, rapidly expanding field of research, providing immense potential in various medicinal applications. The core of this growing area lies in the interaction between light and metallic nanoparticles that turn to plasmonic phenomena. The process is characterized by an external near-infrared (NIR) laser that collectively oscillates free electrons within nanoparticles, producing localized surface plasmon resonances (LSPR), considerably enhancing photon energy conversion efficiency 1. Plasmonic nanoparticles may absorb and convert light energy into localized heating when illuminated at their resonance frequencies. This feature makes them useful for various applications, such as photothermal therapy for cancer treatment, drug delivery, and photodetectors.

The selection of plasmonic metals is critical in harnessing these phenomena. Being a noble metal, Gold is highly inert to biological conditions. Its low cytotoxicity and high photostability make it suitable for PTT applications 2. Semiconducting metal-based nanoparticles (NPs), like magnetic Fe_3_O_4_ NPs 3, molybdenum 4, palladium 5, carbon-based nanomaterials 6,7, or materials loaded with plasmonic photothermal molecules (PPM) such as porphyrin or cyanine derivatives are excellent PPM 8. These NPs (mainly Ag/Au) are frequently combined with chalcogenide semiconductors or orthovanadates to form nanohybrids. Nanohybrid refers to a composite material combining at least two distinct nanoparticles. Combining various nanostructures overcomes the limitations of individual nanoparticles, leading to enhanced properties and achieving multiple functionalities within a single nanoparticle. They have emerged as a promising and innovative diagnostic, imaging and cancer treatment tool. It is critical to understand the structure and composition of these plasmonic materials to tailor their properties for specific applications.

Despite the tremendous potential of PPE, several issues must be addressed. These constraints include concerns about the stability and biocompatibility of plasmonic materials and the necessity for precise heating control in biomedical applications. Overcoming these obstacles while increasing our understanding of plasmonic processes provides new opportunities in nano-therapy. Additionally, regulatory considerations are becoming gradually crucial as plasmonic photothermal technologies evolve. It is critical to ensure the safety of plasmonic materials and devices before they can be successfully implemented in clinical and commercial settings. Regulatory bodies and guidelines (FDA) are evolving to accommodate these innovative technologies and provide a framework for responsible development and application 9.

In exploring plasmonic photothermal effects, we delve into the nanomaterials, challenges, and regulatory landscape, paving the way for a deeper understanding of this field and its transformative potential for treatment.

## 2. Surface-engineered plasmonic nanohybrids for photothermal therapy of cancers

Over the past decades, multimodal therapy has drawn enormous attention to developing precise nanomedicine for cancer. So far, various organic and inorganic nanomaterials have been reported as photothermal agents. However, most nanomaterials suffer from poor biocompatibility and low water solubility. Even though these cannot be retained at the tumor microenvironment for a longer duration, resulting in multi-shots. As a cutting-edge research approach, such photothermal nanomaterials are widely used to develop the functionalized nanohybrid for multimodal therapy towards the precise nanomedicine design. Combining various functionalities in a solitary nanostructure strengthens their unified properties and diverse applications. However, it is a big challenge to develop such a multifunctional nanohybrid platform with desired features like reduced side effects, toxicity, and target-specific release of chemotherapeutic agents sustained to achieve/optimize the antimetastatic therapeutic effectiveness. In this section, we have highlighted promising surface-engineered plasmonic nanohybrids that researchers have developed in the past couple of years to achieve multimodal, synergistic therapy for cancer. We also explained specific upconversion nanoparticles (UCNPs) guided photothermal therapy considering their long-lasting photostability, attractive optical features like narrow emission peaks, and anti-Stokes luminescence under NIR irradiation.

### 2.1. Metallic plasmonic nanohybrids

Some metals containing plasmonic properties are gold (Au), silver (Ag), zinc (Zn), Copper (Cu), Aluminium (Al), platinum (Pt), palladium (Pd), magnesium (Mg), and ruthenium (Rh) 10. Among these metals, gold nanorods are found most promising due to their longitudinal tuneable local surface plasmon resonance (LSPR), biocompatibility, admirable photothermal stability, and high photothermal conversion efficiency (PCE) 11. A synergistic strategy was developed by using dual plasmonic nanomaterials gold nanorods and polyethylene glycol (PEG) functionalized two-dimensional molybdenum disulfide (AuNRs/MoS_2_) nanosheet electrostatically bonded with indocyanine green (ICG) to achieve combined photodynamic and photothermal therapy (PDT/PTT) under low power density (0.2 W/cm^2^) single laser activation. This material was effective in reducing the photobleaching effect of ICG. A unique core-shell nanohybrid platform containing gold nanorods loaded with ICG in the core and coated with mesoporous silica serves as the shell, which is further wrapped with reduced graphene oxide (rGO) attached with polyethylene glycol and doxorubicin (DOX) was developed for maximum anticancer efficiency 12. This study explored the tuneable LSPR of anisotropic gold nanorods (maximum at 758 nm), which was further sifted after mesoporous silica coating (at 768 nm) and loading of ICG (at 780 nm). However, the wrapping with rGO attributed to the 99% quenching of the emission peak of ICG due to the self-accumulation of the dye and the surface energy transfer of nanomaterials. This non-radiating decay, in turn, leads to the photothermal conversion and enhances the photothermal activity of the nanohybrid. This nanohybrid's maximum temperature reached was 61.7º C under 15-minute irradiation of NIR light (808 nm, CW, 2 W/cm^2^). Hence, integrating the multimodal techniques in a single nanohybrid established the ability to detect and treat tumors, drug carrier ability, and significant nano enzymatic activity and photothermal efficacy, showing remarkable tumor specificity. For the first time, a greener nanotechnology strategy hypothesized the design and fabrication of an innovative integrated plasmonic-excitonic nanohybrid consisting of gold nanoparticle (AuNPs) coupled with a layer of semiconductor zinc sulfide quantum emitter (ZnS-QE) surrounded by a macromolecular shell of carboxy methyl cellulose (CMC) 13. In this AuNP-ZnS@CMC nanocomposite, the multifunctional biopolymer CMC simultaneously acting as a biocompatible shell and capping ligand and represented nanotheranostic behavior synchronously by integrating the excitonic activity of ZnS-QE and plasmonic activity of AuNPs. The ZnS-QE layer facilitates the bioimaging, and AuNPs effectively kill one of the most lethal types of brain cancer, i.e., glioblastoma multiforme.

After gold nanoparticles, the silver nanoparticle possesses tuneable surface plasmon strength within a wide biologically transparent window of 650 - 1200 nm, effective light trapping, and intense light scattering potential 14. Folate receptor-targeted quercetin-loaded silver nanoparticles were prepared for photothermal therapy of breast cancer. This innovative pentagonal QRC-FA-AgNPs revealed a robust and tuneable plasmonic field >800 nm region. Upon 5-minute exposure to 1.5 W/cm^2^ NIR light of 808 nm, the hyperthermia effect was potentiated after the quercetin quenching, facilitating targeted endocytosis and enhancing breast cancer cells' thermal sensitivity. An *in-vitro* study on non-small lung cancer cells (NCI-H460) with chitosan-coated silver nano-triangle (Chit-AgNTs) showed a high potential PTT agent in cancer. This nanohybrid, in comparison with thiolated poly (ethylene) glycol-capped gold nanorods (PEG-AuNRs), showed a higher rate of cell destruction upon continuous treatment with Ti: sapphire laser (800 nm) when irradiated at similar laser power density.

Platinum (Pt) is another promising candidate based on its high atomic number and ability to enhance radiotherapy's sensitivity. The maximum absorption of platinum was reported mainly in the ultraviolet region, which lessens their photothermal effect. Despite the potential initiator of merged PTT and chemotherapeutic effect, platinum shows significant cytotoxicity. However, recent studies reported that platinum-based nanocomposites offer good cytocompatibility without significant toxicity. It was hypothesized that a larger number of Pt core aggregations showcases the effective PTT for glioblastoma 15. The ultra-small single-core platinum nano seeds were compared with the multi-core raspberry-like platinum nanoparticles for their PTT on glioblastoma spheroids. Upon irradiation of 808 nm laser with 1.65 W output power, the nano-raspberry exhibited high photothermal conversion in contrast to nano seeds, confirming the fact that multi-core structure mediated by alendronate accumulation within the endosome made them suitable for both intra and extracellular heating leading to death of the cancer spheroids. Packing CeO_2_ into Au@Pt core-shell and further functionalizing with PEG showed the desired targeted photothermal effects along with dual catalytic and peroxidase-like enzymatic activities, which relieve the hypoxia 16.

As a low-cost metal with suitable mechanical catalytic properties and outstanding stability, and considering their green synthesis, palladium nanoparticles are now widely used as PTT agents in cancer therapy. Palladium nanoparticles (PdNPs) functionalized with arginine-glycine-aspartic acid (RGD) enhance its homing at the tumor region by targeting alphaV beta3 (α_v_β_3_) integrin 17. The surface of this nanohybrid was again coated with a low molecular weight water-soluble chitosan oligosaccharide (COS) to improve the biocompatibility of this nanoconstruct (Pd@COS-RGD). This fabricated nanohybrid showed good physiological stability with sustained blood circulation and internalization into the tumor, leading to an efficient 93.4% photothermal conversion at 808 nm laser power and, within 4 minutes, killed 70% of cancer cells. Chaga mushroom-derived anisotropic porous Pd nanoconstruct also exhibited a tri-modal anticancer effect 18. To achieve chemotherapy, DOX was loaded into this porous nanocomposite. Under 4-minute 808 nm NIR laser irradiation in an acidic tumor environment, this nanohybrid sustained release of DOX via electrostatic interaction, resulting in hyperthermic ablation of HeLa cells.

As a transition metal copper nanomaterials mostly, copper sulfide (CuS) has been widely explored in the PTT of cancer owing to NIR absorbing capacity and stimulated reactive oxygen species (ROS) generation ability upon light irradiation. However, considering their toxicity, the release of copper ions is undesirable in our bodies. To overcome this toxicity issue, the copper nanomaterials were functionalized with several polymers such as poly(isobutylene-alt-maleic anhydride), PEG, thiolated PEG, thiolated PEG-COOH followed by reduced graphene oxide modifications 19. These conjugated copper nanohybrids demonstrated enhanced colloidal stability without aggregation for almost a month. However, it was well reported that the NPs of 100-150 nm attributed to enhanced permeability retention effect (EPR), resulting passive accumulation in tumors but unable in trafficking deep solid tumors. Meanwhile, the NPs, like quantum dots with 10 nm or even smaller particle size, easily infiltrate the tumor site, and they are susceptible to hepatic and renal clearance due to their tiny size. To address this drawback, surface-engineered UCNP-guided PTT was developed as an emergent strategy. Zhang et al. designed UCNP@CuS-HA nanocomposite to double-target the explosible nano firework for image-guided deep solid tumor PTT 20. In this study, ~30 nm monodispersed oleic acid-capped UCNPs (NaYF_4_: 2% Er^3+^) were prepared and enveloped with ultrasmall (~4 nm) CuS NPs tethered hyaluronic acid (HA) corona. To facilitate the EPR, the hydrodynamic diameter of the UCNP@CuS-HA nanocomposite was set to 100-150 nm. Improved PTT was observed as the CuS NPs scattered away and infiltrated quickly into the tumor depth and, at the same time, recovered the initial CuS NP-quenched UCNP's luminescence. Wang et al. designed another UCNPs-guided nanohybrid GdOF: Yb^3+^/Er^3+^@(GNDs@BSA)-DOX-FA for targeted image-guided chemo-photothermal tumor ablation 21. UCNPs dominated outstanding NIR-triggered PCE and luminescence properties reported. Simultaneously, GND@BSA was attributed to excellent X-ray attenuation and photothermal ablation. The FA conjugation and BSA coating further reduced the nanohybrids'* in-vivo* toxicity and facilitated their biological circulation. Another review was reported on UCNPs mediated PTT/PDT of cancer with nanocomposites NaGdF4: Yb and Er@Tf-RB. These UCNPs synthesized by thermal decomposition and *in-vitro* anticancer potential were evaluated on 4T1 breast cancer cells 22.

### 2.2. Non-metallic plasmonic nanohybrids

Formerly, the metals were identified as efficient for plasmonic photothermal applications against cancers. However, many of these metals fail to achieve the desired photothermal conversion efficiency due to poor tuneability, radiative loss, and high energy dissipation. As cutting-edge research to overcome such conditions, two-dimensional (2D) materials are progressing with quantum internment effects 23. Some advanced 2D materials are graphene, graphene oxides, metal oxides, non-metals, MXenes, hexagonal boron nitride, and pnictogens. LSPR is not limited to the metallic nanostructures. Semiconductors can show the LSPR effect once they generate adequate carrier concentrations. The non-metallic LSPR nanomaterials have demonstrated additional advantages over the metallic, like scalability, low cost, and high performance 24. The free carrier density of semiconductors is 10^17^-10^22^/cm^3,^ resulting in a broad range of LSPR frequency ranges from THz to near-infrared. Considering the example of graphene, which contains a low level of 2D carrier concentration of around 10^12^/ cm^2,^ resulting in the plasmonic frequency in the middle infrared spectra. Non-metallic plasmonic nanomaterials can be broadly classified as extrinsic doping nanomaterials (doped with heterovalent) and self-doped nanomaterials (deficient anion and cation vacancies) 25. Non-metallic plasmonic nanomaterials overcome their instability, high electron-hole complexation rate, and low carrier concentration using doping, co-crystals, and heterojunctions. These materials can control the free carrier concentration by controlling the composition and concentration of the host material. The titanium carbide (Ti_3_C_2_) nanosheet was reported to be used as a substrate to anchor functional components like nanodrugs and nanozymes. The Ti-based MXene nanocomposites (Ti_3_C_2_T_X_-Pt-PEG) decorated with artificial Pt nanozymes exhibited peroxidase (POD) like activity, which induced apoptosis and necrosis of cancer cells upon irradiation of NIR-II light (at power density of 0.75 W/cm^2^) 26. The temperature elevation from the Ti_3_C_2_T_X_ simultaneously enhanced the POD activity; hence the satisfactory synergistic nanozyme/PTT therapy was established.

#### 2.2.1. Extrinsic doping metal oxide

Metal oxides have demonstrated wide-gap semiconductors, and their energy falls in the visible to near-UV spectrum. To support LSPR, the doping of these materials can be controlled through aliovalent substitutional impurities, interstitial atoms, and the introduction of vacancies, resulting in the generation of sufficient free carrier concentration 27. Many metal oxides doped hetero-valent atoms like Ne-doped TiO_2_, In-doped CdO, Al-doped ZnO, Sn-doped In_2_O_3_ (ITO). Sn-dopped ITO showed excellent electrical conductivity and optical characteristics, opening new scopes in facilitating their application in the MIR and NIR regions. The amount of SN attribute in the changes of LSPR frequencies also enhances the trapping ability 28. Doping metal oxide of AZO reduces the loss of synthesized film through atomic layer deposition and converges a crossover wavelength to near IR with improved optical properties. Subsequent activation is possible with the doped AL in the ZnO matrix. This LSPR mode is exceptionally flexible, varying the doping concentration, ZnO buffer thickness, deposition temperature, heat treatment, etc 29. The introduction of Er^3+^ in the CuS showed the changes in the band gap and defects of the photocatalysts. The photocatalytic performance of this composite was enhanced by introducing the surface defect and Cu vacancies 30. All these examples suggest the influence of doping concentration on the LSPR effect and a tuneable LSPR frequency.

#### 2.2.2. Self-doped metal oxides

Self-doped metal oxides enable LSPR with high-density free carriers. Several materials include ZnO, MoO_3-x_, WO_3-x_, TiO_2-x_, MoS_2-x,_ and copper-deficient chalcogenides 25. Due to the high density of holes in the valance band aroused by the Cu deficiencies, Cu_2-x_S showed excellent plasmonic adsorption in the NIR region. By changing the vacancies in the valency band, the wavelength of the LSPR can be changed 31. Combining more semiconductors (e.g., Zns-CdS-Cu_2-x_S) can form a heterogeneous junction that can widen the light absorption ranges and improve carrier separation efficiency. These heterojunction materials showed wide ranges of spectrum absorption, like UV absorption due to ZnS, visible absorption due to CdS, and NIR absorption due to Cu_2-x_S 32. It was observed that the optical properties and LSPR effect significantly improved this material compared to bare ZnS and binary ZnS-CdS. Synthesized controlled size and shape of Cu_2-x_Te nanoparticles using diphenyl phosphine showed anisotropic growth with cubic structure and better NIR adsorption 33. The ZnS shell of the Cu_2-x_Se@ZnS nanoparticles prevents the oxidation of the Cu_2-x_Se core in a strongly reducing environment. Even slow oxidation still occurred due to the diffusion of Cu^+^ through ZnS, resulting in the LSPR effect in the NIR region due to the Cu vacancies 34.

Oxygen vacancy defects are another crucial parameter to enrich the photocatalytic activities and light capturing of metal oxides, resulting in the light region extension to NIR. A few examples of this category are ZnO, MoO_3-x_, TiO_2-x_, and WO_3-x_. These materials show strong oxidation properties, are non-toxic, and have low manufacturing costs. WO_3_ can be synthesized by different methods to improve the photolytic activity. The photocatalytic efficiency was enhanced significantly by introducing O_2_ vacancies in this molecule. Additionally, absorption in the visible light and conductivity can be improved by the LSPR effect of AO_3-x_ in the NIR region. Oxygen vacancy was introduced at low-temperature annealing in alcohol to WO_3_ to get WO_3-x,_ resulting in a high concentration of oxygen vacancies. That leads to the recombination of photogenerated electron-hole pairs, improvement of separation efficiency of free carriers, and photocatalytic performance 35. W_18_O_49_ showed an effective broad-range absorption of NIR and visible light antenna to modulate a full spectrum solar-light-driven photocatalysis 36. This LSPR is generated from the localized electron confinement around the lattice W^5+^-W^5+^ pairs of the structure of W_18_O_49_. Achieving optimum photocatalytic activity and prerequisite characteristics like adsorbing light across the whole solar spectrum and generating active charge by a single semiconductor with oxygen vacancies is difficult. The instability problem of plasmonic photocatalyst WO_3-x_ in an aqueous solution was achieved by introducing photoinduced electron injection to construct CdS/WO_3-x_ heterostructure 37. These non-elemental metal plasmonic nanowires are more stable and active than the WO_3-x_ and semiconductor CdS. MoO_3_ is another non-toxic and low-cost semiconductor that shows optimum adsorption capacity. The light absorption range was broadened when oxygen vacancies were introduced to the MoO_3_ to develop MoO_3-x_. This material also forms complexes with other semiconductors to improve the photocatalytic activity. The heterojunction interaction between MoO_3-x_ and CdS initiates interfacial charge transfer, migration of photoexcited charge carriers, and charge segregation by reducing the electron-hole pairs 38. CdS/MoO_3-x_ is a novel photocatalyst with MoO3-x and varying proportions of CdS nanospheres. During the synthesis, polyvinylpyrrolidone was a reducing agent that generated oxygen defect in MoO3 and helped develop crosslinking between CdS and MoO_3-x_. This composite demonstrated higher visible light photocatalytic performance. In addition, hole-oxidized photo corrosion of CdS was suppressed due to the presence of the hole-attractive MoO_3-x_.

#### 2.2.3. Plasmonic metamaterials

Metamaterials open a new science scope comprising nanoscience, optics, physics, material science, and engineering. The overexpressed interest in these materials is mainly due to their building blocks' unique internal physical structures 39. These are three-dimensional macroscopic composites and periodic cellular architecture designed to show specific excitation. Including slight inhomogeneities can produce a distinct, effective macroscopic behavior 40. Earlier researchers focused either on plasmonic or metamaterials rather than the outstanding features and responses originating from the interplay between these two materials. It is well demonstrated that combining these two materials could open up a wider opportunity and establish a new cross-disciplinary approach in the PTT of cancer. Graphene has gained attraction as a plasmonic metamaterial owing to its unique electronic, mechanical, optical, and thermal properties 39. Combining plasmonic and graphene physics could create a versatile platform for photothermal application in optical and terahertz regimes.

Mesoporous silica-coated gold nanorods (AuNRs), a dual responsive nanohybrid, were developed for triple-combination therapy of breast cancer 41. Doxorubicin and IR 820 photosensitizer were co-loaded into the degradable silica pores. Hyaluronic acid (HA) was encapsulated into the nanocomposite to endow it with improved biocompatibility for targeting mammary carcinoma. After endocytosis, this nanohybrid was degraded rapidly by hyaluronidase (HAase) and glutathione (GSH), releasing IR 820 and DOX into the tumor site. Irradiation of 808 nm laser on this nanohybrid triggers the photothermal response and shows profound photodynamic and chemotherapeutic activity, leading to a highly efficient antitumor effect.

## 3. Limitations of plasmonic nanohybrids

A significant obstacle in translating plasmonic nanoparticles (P-NPs) into clinical practice is the inadequate accumulation of P-NPs in the targeted tissue. It was hypothesized that these P-NPs should demonstrate good solid tumors enhanced permeability and retention (EPR) effect. However, agglomeration and stability pose potential obstacles to their performance and further applications. P-NPs often exhibit a high EPR effect due to their small size and large surface-to-volume ratio, leading to agglomeration due to NPs sticking. This agglomeration reduced the stability of P-NPs and hindered their long-term storage 42. Several other factors influence the agglomeration of P-NPs, including their size, shape, surface chemistry, and concentration. Smaller P-NPs exhibit a higher surface area-to-volume ratio, making them more susceptible to agglomeration than larger P-NPs. Additionally, P-NPs with sharp edges or corners are more prone to agglomeration than those with smooth surfaces. The surface chemistry of P-NPs also plays a crucial role. Hydrophilic P-NPs tend to agglomerate less than hydrophobic P-NPs. Finally, the concentration of P-NPs in a solution directly impacts agglomeration. Higher concentrations of P-NPs have a greater likelihood of agglomerating compared to lower concentrations. These factors collectively influence the agglomeration behavior of P-NPs and must be carefully considered when designing and utilizing P-NPs for biomedical applications 43. Agglomeration can have several adverse effects on the performance of P-NPs. For example, agglomeration can reduce the surface area of P-NPs, which can reduce their catalytic activity towards targeted tissue and other surface-dependent properties.

Additionally, agglomeration can reduce the light scattering efficiency of P-NPs, which can hinder their use in optical applications 44. To prevent the agglomeration of P-NPs, a common approach is to coat the P-NPs with a stabilizing agent. Stabilizing agents can prevent the P-NPs from sticking together by electrostatically creating a steric barrier or repelling each other. Another approach to preventing agglomeration is to functionalize the surface of the P-NPs with hydrophilic groups, which can make the P-NPs more water-soluble and less likely to agglomerate 45. Limited light absorption is a foremost challenge for P-NPs in plasmonic photothermal therapy (PPTT). The effectiveness of PPTT depends on the ability of P-NPs to absorb light, and P-NPs with limited light absorption in specific wavelength ranges will be less effective for this therapy.

Several factors contribute to the light absorption of P-NPs, including size, shape, composition, and surface chemistry. Smaller graphene oxide-gold nanorods (GO-AuNR) have broader absorption bands than larger GO-AuNR. Additionally, GO-AuNR with different shapes and compositions can have different absorption bands. Finally, the surface chemistry of P-NPs can also affect their light absorption.

To improve the light absorption of GO-AuNR for PPTT, researchers are designing particles with specific sizes, shapes, and compositions that match the wavelength of light that will be used for treatment 46. Another approach is to coat P-NPs with a material that can enhance their light absorption. For instance, Antibody-conjugated single-walled carbon nanotubes (SWCNTs) are a promising new class of photothermal therapy (PTT) agents. They effectively kill cancer cells when illuminated with near-infrared (NIR) light. The SWCNTs are coated with a gel that is conjugated with antibodies that are specific to cancer cells. When the SWCNTs are illuminated with NIR light, they absorb it and convert it into heat. This heat kills the cancer cells. The antibody-conjugated gel coating enhances the light absorption of the SWCNTs, making them more effective at killing cancer cells 47. Gold nanorods (AuNRs) with low light absorption in the NIR region are less effective for plasmonic photothermal therapy. Still, they can penetrate deeper into tissues, making them suitable for treating deeper tumors beneath the skin.

The therapeutic window is a critical parameter in PPTT, representing the range of light wavelengths that can be safely employed without causing damage to healthy tissues. This therapeutic window is typically narrow for plasmonic nanohybrids, often restricted to the near-infrared (NIR) spectrum (700-1000 nm). The absorption spectrum of these hybrids is influenced by their composition, size, and shape. Sometimes, the absorption spectrum may be narrow, limiting their effective light absorption to a restricted wavelength range. That poses challenges for clinical applications, as finding a wavelength of light that effectively eliminates cancer cells while safeguarding healthy tissues can be problematic.

Multiple factors contribute to the narrow therapeutic window for metal-semiconductor hybrids in PPTT. One factor is the strong light scattering exhibited by these hybrids. They can disperse light over a broad range of wavelengths, potentially leading to excessive heating and damage to healthy tissues. Another factor involves the nonlinear optical properties of these hybrids. They can absorb light at one wavelength and emit it at a different wavelength. That can result in heat generation at wavelengths not absorbed by the hybrids, further jeopardizing healthy tissues.

Gold-silicon (Au-Si) nanorods exhibit a limited absorption spectrum within the NIR region (700-1100 nm), making finding a wavelength that effectively eliminates cancer cells while safeguarding healthy tissues is challenging. Researchers are exploring strategies to modify the composition and structure of Au-Si nanorods to broaden their absorption spectrum and expand their therapeutic window 48. In addition to potential toxicity, other concerns are associated with using inorganic nanoparticles (such as Au NPs) in PPTT, including their distribution in the body, long-term effects, and clearance. Differences in the size, shape, structure, and surface properties of nanoparticles, combined with specific characteristics of cells and tumors *in vivo* models, create a complex challenge in fully understanding and addressing toxicity issues. Positive results from early preclinical studies often involve *in vivo* cellular models and subcutaneous xenograft murine models. Still, these don't precisely mirror the complexities of cancer problems and clinical conditions. The advancement of PPTT is essential to develop more accurate *in vivo* and *in vitro* models considering the diverse heat responses and temperature regulation mechanisms of various cell cultures and animals 49. Certain core-shell hybrids may exhibit cytotoxic effects or trigger immune responses, limiting their therapeutic potential. For instance, gold-silica (Au-SiO_2_) core-shell nanoparticles have been shown to induce apoptosis and cell death in certain cell lines 50, while silver-silica (Ag-SiO_2_) core-shell nanoparticles can elicit inflammatory responses 51.

Core-shell hybrids' cytotoxic and immunogenic properties can lead to adverse effects, such as liver damage, kidney dysfunction, and systemic inflammation. Therefore, it is essential to carefully assess core-shell hybrids' biocompatibility and toxicity profile before clinical application in PPTT. The effectiveness of PPTT can be limited by the limited penetration depth of light in biological tissues. This limitation is particularly relevant for nanoporous hybrids like mesoporous silica nanoparticles (MSNs) located deep within tissues, as they may not receive sufficient light for effective heat generation. For instance, near-infrared (NIR) light, commonly used in PPTT, can penetrate tissues only 2-3 millimeters. Consequently, MSNs beyond this depth may not generate enough heat to kill cancer cells 52.

Researchers are exploring strategies to enhance light penetration depth to address this challenge, such as using longer wavelengths of light. Near-infrared (NIR) light with 650-950 nm wavelength has been shown to penetrate tissues deeper than visible light. For instance, NIR light with a wavelength of 800 nm can penetrate tissues up to 5 mm, while NIR light with a wavelength of 1064 nm can penetrate tissues up to 10 mm 53. The use of longer wavelengths of NIR light has been shown to improve the efficacy of PPTT in animal models. For example, a study by Zhou et al. showed that PPTT using Molybdenum disulfide (MoS_2_) nanodots with a NIR absorption peak of 1064 nm was more effective at killing cancer cells in mice than PPTT using hyaluronic acid Molybdenum disulfide HA-MoS_2_ nanodot with a NIR absorption peak of 808 nm 54.

Developing nanoporous hybrids with improved light scattering properties could further enhance their ability to absorb and utilize light for effective PPTT. The nanoarray of nanoporous hybrids can trap light by enhancing the scattering effect and prolonging the effective transmission channel. This results in good light absorption, essential for photothermal treatment 55. The light propagation dynamics within biological tissues embedded with nanoparticles are intricately linked to the anisotropic scattering properties of the incorporated nanoparticles. These properties are demonstrably influenced by a quartet of factors: nanoparticle morphology, dimensions, orientation, and incident light wavelength. Crucially, nanoparticles tending to forward scattering behavior facilitate enhanced light penetration depth. This phenomenon translates to a more uniform distribution of both the Specific Absorption Rate (SAR) and temperature within the tissue, a pivotal factor in achieving precise thermal damage localization during therapeutic applications 56. Gold nanoparticle-coated mesoporous silica nanoparticles (MSNs) and a silica shell have enhanced light-scattering properties. Gold nanoparticles can act as nano-antennas due to the gold core for visible and infrared radiation, enhancing the interaction of light with nanoscale matter and concentrating light onto the MSN 57. As a result, gold nanoparticle-coated MSNs can absorb and utilize light more effectively than uncoated MSNs 58. Carbon nanotubes (CNTs) are also known for their light-scattering properties. MSNs coated with CNTs have enhanced light-scattering properties, which can improve their ability to absorb and utilize light for PPTT 59. In addition to these examples, researchers are continuously developing new nano-porous hybrids with improved light-scattering properties. These hybrids have the potential to revolutionize the field of PPTT and provide new and more effective treatments for cancer and other diseases.

Heterogeneity and variability in the size, shape, and composition of plasmonic NPs like metal-semiconductor hybrids can significantly impact their photothermal properties and therapeutic outcomes in PPTT. Achieving consistent and reproducible results can be a challenge due to these variations. For instance, gold-silicon (Au-Si) nanorods with different aspect ratios exhibit distinct light absorption and heat generation capacities, affecting their therapeutic efficacy 60. Similarly, SiO_2_-coated silver (SiO2@AgNPs) nanoparticles with varying shapes and sizes display diverse optical properties, influencing their photothermal performance 61. These inconsistencies in metal-semiconductor hybrids can lead to unpredictable treatment outcomes, hindering their clinical translation in PPTT. Therefore, developing strategies to synthesize metal-semiconductor hybrids with uniform size, shape, and composition is crucial for achieving reproducible photothermal properties and enhancing the efficacy of PPTT. Table 1. Summarizes the limitations of above explained nanohybrids.

## 4. Green synthesis of plasmonic nanoparticles way to future directions

**Figure 1:**
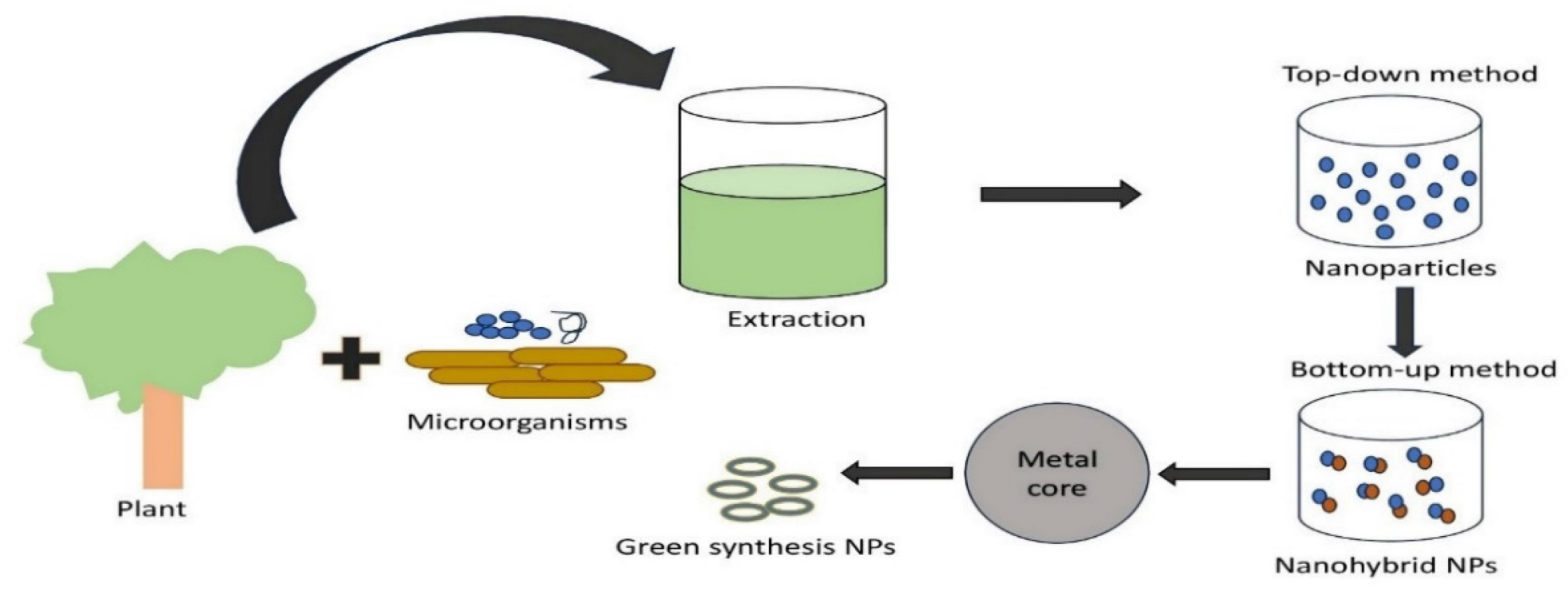
Simple process of green synthesis plasmonic nanohybrid nanoparticles 65-70.

The numerous uses of plasmonic nanoparticles (PNPs) in nanotechnology have increased the need to develop new synthesis techniques. Consequently, several protocols have been developed for synthesizing PNPs in different sizes, shapes, and compositions (Figure 1). In addition to microorganisms like bacteria, algae, fungus, and yeast in the known green synthesis methods of today (Table 3), metal ions are bio-reduced to their corresponding nanomaterials using plants or plant components (Table 2) and informational macromolecules like proteins, polypeptides, DNA, and RNA. The potential of biogenic nanoparticles for cancer nanomedicine was demonstrated by their rapid internalization by cancer cells, affecting their cellular organization and karyoplasmic ratio 64,65.

The study examined the cytotoxic effects of *Indigofera tinctoria* leaf extract and nanoparticles on the A549 lung cancer cell line 71. It has been demonstrated that when concentration increases, cell viability decreases and that nanoparticles are more harmful to cancer cells than pure leaf extract. The goal of creating gold and silver nanoparticles was to treat breast cancer cells by imparting antioxidant, catalytic, and antimicrobial properties using an aqueous extract of dried *Amomum villosum* (cardamom) fruits 72. The process was done at room temperature. Gold nanoparticles from the leaf extract of *Sasa borealis* were used. These gold nanoparticles were found to have anticancer activity against gastric adenocarcinoma cancer (AGS) cells and have a toxic effect on normal and embryonic kidney cells (HEK293). Within the Poaceae family of grasses is the bamboo species *Sasa borealis* 73. Its reported anticancer activity. The MTT assay demonstrated that AuNPs cause toxicity based on the dose-dependent growth inhibition of A549 cells 71. Rat basophilic leukemia (RBL) cell monolayer was used using the scratch assay, and the silver nanoparticle's anticancer potential was assessed. The outcomes demonstrated that the synthetic silver nanoparticles hindered RBL cell migration. The produced silver nanoparticles demonstrated antimicrobial potential with minimum inhibitory concentration (MIC) values ranging from 4-16 µg/mL against strains of bacteria that are both Gram-positive and Gram-negative 74. Reactive oxygen species (ROS) can be produced by zinc oxide (ZnO) nanoparticles when they encounter light and have good photocatalytic qualities. When the ZnO NPs are exposed to light, they release ROS, which can provide protection from harm to healthy tissues and specifically destroy cancer cells through oxidative stress 75. A high toxicity effect was observed at concentrations of 4.7 μg/mL-1, as discovered by testing the in vitro cytotoxicity of various concentrations of flaxseed-derived iron oxide nanoparticles (IONPs) against MCF-7 cells. *Psoralea corylifolia*-mediated IONPs have been shown to have a remarkable cytotoxic effect against renal tumor cells, as well as potential anticancer activity 76. Copper nanoparticles (CuNPs) and an aqueous extract from *Allium noeanum* leaves 77. Copper nanoparticles have been shown to have anti-human endometrial cancer effects against HEC-1-A, HEC-1-B, Ishikawa, HepG2 cancer cells, and KLE cell lines exhibiting high cell death. Using Abutilon indicum leaf extract, which is non-toxic, simple, and inexpensive, the hierarchical CuO NPs were created using a green chemistry method 78. The greenly synthesized Cr2O3 nanoparticles demonstrated significant antioxidant and anticancer effects on MCF-7 cancerous cells and the linoleic acid system 79. The findings show that the platinum-palladium nanoparticles (Pt-PdNPs) exhibited notable cytotoxic effects against the lung cancer A549 and the breast adenocarcinoma MCF-7 cells, with IC_50_ values of 8.8 and 3.6 µg/mL. These values are analogized with those of PtNPs 10.9 and 6.7µg/mL, PdNPs IC_50_ 31 and 10.8µg/mL, and carboplatin IC50 23 and 9.5µg/mL, respectively 70,80.

Silver nanoparticles (AgNPs) were green synthesized using the extracellular methodology and *Pseudoduganella eburnea* MAHUQ-39. This process is easy, inexpensive, and environmentally friendly. *Pseudoduganella eburnea* MAHUQ-39 is a bacteria identified from a soil sample 90. AgNPs were synthesized effortlessly and environmentally without the necessity for reducing agents using the culture supernatant of *Pseudoduganella eburnea* MAHUQ-39. Additionally, the antibacterial efficacy of environmentally synthesized silver nanoparticles (AgNPs) was examined against human pathogens that exhibit resistance to multiple drugs. Several microorganisms have been observed recently, including Novosphingobium species, Brevibacterium frigoritolerans, and Bacillus methylotrophicus. For environmentally friendly silver nanoparticle synthesis, THG-C3 has been isolated. After being exposed to varying concentrations of AgNPs. They demonstrated antibacterial activity (MBC 64-256µg/ml^-1^ and MIC 8-128µg/mL^-1^) and significantly reduced ATP levels in bacterial cells. AgNPs were discovered to be cytotoxic to MCF-7 breast cancer cells and RAW 264.7 macrophages in a dose-dependent manner, with the latter type of cells exhibiting higher cytotoxicity than the former following *in-vitro analysis*. The green AuNPs showed potent cytotoxicity against MCF-7 cancer cells, acceptable levels of cytotoxicity against normal cells, and enhanced activity against pathogens 94. The outcomes show that using AuNPs produced by *Anacardium occidentale* (Leaves) for antibacterial purposes is safe.

## 5. Preclinical challenges

Although the development of PPT is a unique method for selectively targeting and eliminating cancer cells while sparing healthy tissue, considerable preclinical obstacles persist. These agents must have certain qualities, such as significant NIR absorption, thermal stability, and ideal physicochemical parameters, such as size, shape, biocompatibility, and stability in biological fluids. One major obstacle involves effectively delivering photo-absorbing agents to the tumor site, which requires navigating rigid barriers, including the impermeable blood-brain barrier. Simultaneously, ensuring the precise delivery of NIR light irradiation to the tumor site requires overcoming biological impediments without causing unintended damage. Many *in-vitro* studies required laser power of more than 6 W/cm^2^ 95,96. However, in a study, glioblastoma mice were treated with NPs camouflaged with brain metastatic tumor cell membranes. This study displayed that 1.0 W/cm^2^ of laser power was sufficient to effectively decrease tumor development and penetrate the blood-brain barrier 97. Maintaining strict control over power density within safety limits (1.0 W/cm^2^ and 0.33 W/cm^2^ for 1,064 and 808 nm lasers, respectively) and achieving sufficient light penetration depth for inducing localized hyperthermia (HT) is of paramount importance 98.

Yu He et al. investigated the risks of PTT in brain tissue. Following laser therapy, they examined temperature variations in three distinct porcine brain tissues. They discovered that the optical and thermal characteristics of different tissues differed. Hyperthermia was established with the injection of gold nanoshells (AuNSs) and silver nanoplates (AgNPs); however, the effect was attenuated due to substantial absorption and scattering of brain tissue. The loss of laser intensity was measured as it passed through the cerebral tissues 99.

PTT-PDT combination therapy has shown remarkable potential for cancer therapy 100,101. However, having a spectral incompatibility between a PDT agent and a PPM, sequential irradiation with two separate lasers is needed to activate PDT and PTT. This results in a lengthy and challenging treatment period 102,103. Furthermore, aligning both laser beams in a single place is challenging. To initiate synergistic PDT/PTT or even single-mode PTT, a high-power laser (1 W/cm^2^) irradiation is required. However, it poses safety issues because it tends to cause skin burns. Due to the limited PTT performance of a single PTT agent, a significant amount of PTT agent is usually placed under long-term laser irradiation to induce localized hyperthermia, which may cause damage to normal tissues. To achieve a simultaneous synergistic PDT/PTT effect under single low-power NIR laser irradiation, an AuNRs/MoS_2_ hybrid was constructed, which could generate extreme heat under a single power of 0.2 W/cm^2^ 104.

Developing nanomaterials with a strong absorption in the NIR-I region is also desirable. One such nanohybrid was developed by Qui et al. Au@MgFe_3_O_4_ improved NIR absorption while providing magnetic characteristics for MRI-guided cancer cell PTT. A scab was noticed on the epidermis, implying burns resulting from PTT-generated heat 105. Laser irradiation was performed in time intervals to avoid tissue injury from hyperthermia. In the first interval, the surface temperature of the tumor increased from 34.7 to 53.8 °C, reaching 54.8 °C in the second. It was found to have the ability to kill cancer cells. The epidermis was not burned, and the temperature dropped to body temperature within 1 minute, indicating a safer therapy 12.

Until now, most research focused on generating NIR-I (700-900 nm) triggered nanohybrids. However, the intrinsic advantages of NIR-II (1000-1700 nm), like deep penetration into tissues, precise spatial resolution, and high maximum permissible laser exposure to tissues, surpass those with plasmonic characteristics in visible (400-700 nm) and NIR-I. These NIR-II plasmonic materials, in particular, could be used for in vivo visualization of deep tumors 106, 107. All the above studies have been summarised in Table 4.

## 6. FDA regulatory aspects

An image-guided study using anisotropic plasmonic gold nanorod-Indocyanine green@reduced graphene oxide (GO)-doxorubicin nanohybrids demonstrated enhanced tumor theranostic. Indocyanine green (ICG), which has FDA approval, has shown a great deal of promise when combined with various nanoplatforms for near infra-red (NIR) contrast imaging and Photothermal therapy (PTT) or photodynamic therapy (PDT) 12. ICG is an effective NIR-absorbing PTT agent with remarkable light-to-heat conversion efficiency for cancer treatment. A pilot clinical study conducted in 2011 showed the potential intervention of ICG for metastatic breast cancer. Ten patients were enrolled in this study with advanced-stage metastatic breast cancer received. Laser immunotherapy was given by local injection of ICG and glycated chitosan, followed by 805 nm laser irradiation at a power density of 1 W/cm^2^. This therapy achieved an objective response rate of 62.5% and a clinical benefit response rate of 75% 115.

Now, the FDA has approved a few nano-based medications, including Caelyx, Dolix, Abraxane, and Transdrug. In addition to serving as a model drug carrier as a nano vehicle for cancer therapies, ultrathin graphene oxide (GO) is a promising option for enhancing the stability and effectiveness of hybrid nanostructures for various applications 116,117. NIR laser-induced photothermal therapy (PTT) converts optical energy into thermal energy through a photothermal agent, and it can potentially be an effective localized, minimally invasive antitumor treatment. The FC-808 fiber-coupled laser system works at a wavelength of 808 nanometres and has a power of 0.5 W/cm^2^. It was used to perform NIR laser-induced photothermal heating 118. By embedding ICG on mesoporous silica-coated gold nanorods (GNRs) and wrapping reduced graphene oxide (rGO) 12, in addition to attaching DOX and polyethylene glycol, A hybrid material that was smaller than 100 nm was produced. The hybrid material also possesses three remarkable properties: nonenzymatic activity, photothermal activity, and drug carrier ability. The biological tumor detection and therapy window was appropriate for its NIR absorption capability, approximately 780 nm. Discoveries from both *in-vitro* and *in-vivo* experimentations recommended that administering GNRS-ICG@rGO-DOX hybrid material under 808 nm laser irradiation could effectively suppress the growth of HT-29 tumors 69,119. Combining photothermal therapy techniques and chemotherapy by inducing cell apoptosis with the anticancer drug DOX and catalytic therapy by producing excess reactive oxygen species (ROS) in the tumor location causes apoptosis mediated by mitochondria 120-125.

It was recently discovered that doxorubicin could be found as a therapeutic payload in hybrid micellar nanoparticles with multiple functions that include metal NPs for magnetic resonance imaging (MRI), near-infrared fluorescent imaging with quantum dots, prolonged circulation times with polyethylene glycol (PEG), and F3-peptide specific to tumor 90,116,125. FDA-approved Doxil- and Doxo- 125 liposomal formulation as anticancer nanotherapeutic. Auranofin is an FDA-approved triethyl phosphine that contains gold 126. It is used to treat rheumatoid arthritis, cancer, neurodegenerative diseases, HIV/AIDS, parasitic infections, and bacterial infections 124,126. Recently, gold-on-gold homometallic hybrids with controllable overgrowth of either spherical or branched gold domains on the GNR surface were obtained, along with the interface energy and growth kinetics. Under the NIR-II laser, pairwise plasmonic GNR@Cu2-x, these heterostructures might be used for photothermal ablation of cancer cells *in-vivo* and *in-vitro*. The growth of tumors is significantly inhibited when the GNR@Cu2-xSe heterostructures and NIR-II laser are combined 106. The physiochemical properties, antiviral, anticancer, scalability, biocompatibility, and cost-effectiveness of metal nanoparticles, especially those formulated through green nanotechnology or green chemistry, have attracted a lot of interest as cancer or viral therapeutics and therapeutic drug delivery systems 124.

Aurolase ® is a 150 nm gold nanoshell based on another PTT system that Nanospectra Biosciences developed. It contains a 120 nm silica core as the dielectric core, a 15 nm gold shell for NIR light-responsive thermal ablation, and a polyethylene glycol layer 124. Another emerging technology, AuroShell ® (also developed by Nanospectra Biosciences), demonstrated better accumulation in tumor tissue through enhanced permeability and retention effect. AuroShell particles comprise a 10-20 nm thick gold shell deposited on a solid silica (silicon dioxide) core. These particles do not accumulate in healthy tissue and are cleated by the reticuloendothelial system from the bloodstream. These particles were coated with a 5000 molecular weight methoxy polyethylene glycol (mPEG) chain through the thiol bond to stabilize in saline solution. After administration, this coating improved stability and enhanced the circulating half-life 127. Another clinical trial study was reported on nanohybrid Auroshell containing silica core and gold nanoshell of ~ 150 nm diameter to treat and ablate prostate cancer 128. This study was conducted on sixteen patients. During the intravenous infusion of the gold silica nanoshell (GSN), one patient experienced transient epigastric pain and, hence, was excluded from the study. The median number of laser excitations required was 25. However, the laser power was also increased from 4.5 W to 6.5 W to expand the laser ablation zone. As a positive outcome, this pilot study complied with the safety end point according to common terminology criteria for adverse events (CTCAE) during 90 days of follow-up. 60% cancer-free ablation zones were reported in 9 patients out of 15 at three months of the therapy. In contrast, 87% of cancer-free zones were reported in 13 patients out of 15 at 12 months of the therapy. The outcome of this clinical trial study demonstrated that GSN-directed laser-induced ablation is a technically adequate, safe, and feasible procedure that holds promise for targeted tumor destruction without causing collateral damage to the surrounding healthy tissues and organs. The above explained clinical trial study summarized in Table 5.

## 7. Conclusion and prospects

It is essential to check the uniformity and reproducibility before the production scale-up of these plasmonic nanohybrids. According to the recent plasmonic materials market forecast, Asia Pacific is expected to account for the largest share from 2023 to 2031. Day-by-day, the oncological drug approval and reaching the clinical phase increases significantly. Despite much struggle, very small proportion of plasmonic photothermal therapy reached the clinical phase. That is mainly due to the lack of safety, formulation prospects, and effective delivery. All these challenges need the development of new clinical protocols and translation methods. Another promising drawback is the long-term toxicity of PPTT. The surface-engineered and conjugated nanohybrid developed through synthetic or green technology needs to have good bio- and hemo-compatibility. At the preliminary stage, animal experimentations are done on the lower rodents, which is quite distinct from human trials 129. The long-term toxicity study should not be restricted to six months as this period is insufficient to predict the potential toxicity of PPTT. Delivery of metal or non-metal plasmonic nanomaterials to the target tumor site is crucial for effective therapy, reduced side effects, and less adverse effects on the healthy tissues.

Active and passive mechanisms mainly achieve drug targeting. During passive targeting, the particle size (50-200 nm) is crucial in accessing and penetrating cells. Active targeting can also be made possible by embedding ligand conjugation with the plasmonic nanomaterials. Synthesizing plasmonic nanohybrid through green synthesis opens a new scope with improved cytotoxicity. More focus must be emphasized on the environmentally friendly green synthesis method for plasmonic nanohybrids. The scaling of nanohybrids to industrial scale should be considered during the design of new synthetic methods. Considering the tremendous advancement of plasmonic and metamaterials assembled, nanohybrid will inevitably offer promising opportunities to address the grand challenges in the PTT of cancers. However, the penetration of NIR light through human tissues is limited to a few depths, and delivery of non-ionizing radiation to well-defined target tissue volumes is challenging. These results in incomplete access to tumor cells and ablative modalities 130. Recently, specific UCNPs-based multimodal imaging probes have emerged as sophisticated technology in biomedical theranostic applications aimed to fulfil effective cancer therapy by enabling deep tumor penetration, activating the caspase-induced cancer cell apoptosis, NIR-triggered enhanced ROS generation, and profound photothermal conversion.

Additionally, the combination of photothermal therapy with chemotherapy can be an effective treatment option for controlling cancer. In combination, chemotherapy may elicit the efficacy of PTT by inhibiting the regrowth of damaged tumor blood vessels. Integrating nanoparticles with photosensitizing agents and drugs opens a new direction for future research that can be validated through clinical testing.

## Figures and Tables

**Table 1:** Limitations of plasmonic nanohybrids used in photothermal therapy of cancer

Nanohybrids	Limitations	Reference
Gold	It is difficult to synthesize with high purity and reproducibility, and they can be susceptible to photodegradation. Additionally, some gold nanohybrids can be cytotoxic.	62
Silver	It isn't easy to synthesize with high purity and reproducibility, and they can be less stable than other plasmonic nanohybrids. Silver nanohybrids can be more challenging to target specific cells and tissues.	51
Graphene	Limited light penetration depth: This can make it challenging to ablate large tumors or tumors located deep inside the body. Additionally, graphene nanohybrids can be more susceptible to aggregation and sedimentation.	63
Silicon	A limited absorption spectrum in the NIR region can make them less effective for PTT. Additionally, silicon nanohybrids can be more difficult to synthesize with high purity and reproducibility.	48
Molybdenum	A limited absorption spectrum in the NIR region can make them less effective for PTT. Additionally, molybdenum nanohybrids can be more difficult to synthesize with high purity and reproducibility.	54

**Table 2:** Green synthesis plasmonic nanohybrids extracted from plants

Plasmonic material	Plant	Cell line	Shape and size of nanohybrid	References
Gold (Au)	*Indigofera tinctoria*	A549 lung cancer cell lines	Spherical, triangular, and hexagonal;16-46 nm	71
	*Sasa borealis*	Normal cells HEK293 and AGS cells	spherical, oval; 10-30 nm.	73
	*Marsdenia tenacissima*	A549 lung cancer cell line underwent induced apoptosis by altering the Bax/Bcl-2 protein levels.	Spherical and oval-shaped; 40-50 nm	71
	*Brazilian Red Propolis*	Bladder (T24) cancer cell and Prostate (PC-3) cancer cell line	Spherical; 8-15 nm	81
	*Abies spectabilis*	Bladder cancer (T24) cell line	Spherical; 20-200 nm	82
	*Stevia rebaudiana*	PC-3 and MCF-7 cancer cell line	Spherical; 15-25 nm	83
Silver (Ag)	*Amomum villosum/ E. cardamomum*	Breast cancer cells (MCF-7)	Spherical; 5-15 nm	72
	*Mukia maderaspatna*	MCF-7 cells	Spherical, triangle, and hexagonal; 20-50 nm	84
	*Pandanus odorifer*	RBL cancer cells	Spherical; 10-50 nm	74
Zinc Oxide (ZnO)	*Moringa oleifera, Eclipta alba, Lycopersicon esculentum*	HeLa cell line	Agglomerated; 60 nm	75
Iron oxide (ION)	*Psoralea corylifolia*	MCF-7 cell and Renal tumor cell	Spherical; 25-30 nm	76
	*aloe vera*	MCF-7 breast cancer cell	Regular sphere; 50 nm	85
Copper (Cu)	*Allium noeanum, Nerium oleander, Eclipta prostrata, Magnolia Kobus*	HepG2 Cancer cell line	Spherical; 50 nm	77,86
	*Abutilon indicum*	A549 lung and MDA-MB-231 Brest cancer cells	Spherical; 10-30 nm	78,87
	*Sargassum polycystum*	MCF-7 breast cancer cells	50-60 nm	
Chromium oxide	*Abutilon indicum*	MCF-7 cell	17-42 nm	79
Palladium and Platinum (Pd-Pt)	*Dioscorea bulbifera*	HeLa cancer cell	Spherical; 10-25 nm	80,88
	*Evolvulus alsinoides*	Human ovarian A2780 cancer cells	Spherical; 5 nm	
	*Peganum harmala*	Lung cancer cell A549 and Breast MCF-7	11-12 nm	70

**Table 3:** Green synthesis plasmonic nanohybrids extracted from microorganisms

Plasmonic materials	*Microorganisms*	Cell line	Shape and size of nanohybrid	References
Gold (NPs)	*Bacillus subtilis, Escherichia coli*	Brest cancer cell line (MCF-7)	Spherical; 10-30 nm	70
	*Escherichia coli and Bacillus subtilis*		Spherical; 10-50 nm	89
Silver (Au)	*Pseudoduganella eburnean* and *Staphylococcus aureus*	MAHUQ-39	Spherical; 8-24 nm	90
	*Bacillus methylotrophicus, Brevibacterium frigoritolerans, Novosphinggobium species*	THG-C3		
	*Escherichia coli, Klebsiella pneumoniae, Pseudomonas aeruginosa, Staphylococcus aureus*	MCF-7	Spherical; 13 nm	91
	*Bacillus subtilis* and* Staphylococcus aureus*	Human colon cancer (COLO205), and human prostate adenocarcinoma (LNCaP) cell	20-50 nm	67,84
Palladium (Pd)	*Staphylococcus aureus* and* Escherichia coli*	RBC	27 nm	70
		Human cervical cancer cells (HeLa)	10-25 nm	
	*Escherichia coli* and* Pseudomonas aeruginosa*	A2780 cancer cell	10-20 nm	92
	*Staphylococcus aureus* and* Bacillus subtilis*	A549 lung cancer cells	50-150 nm	88
Chromium oxide (Cr2O3)	*Escherichia coli, Staphylococcus aureus, Bacillus bronchiseptica,* and* Bacillus subtilis*	MCF-7	1-100 nm	79
Copper	*Pseudomonas aeruginosa* and* Aspergillus niger*	MCF-7	10-20 nm	87
	*Escherichia coli* and* Pseudomonas mirabilis.*	A549	20 nm	93

**Table 4:** Comparison between various nanohybrids for photothermal therapy of cancer.

Nanohybrid	Size and morphology	Cell line used	Animal used and treatment condition	Laser (Power, Time); Photothermal conversion efficiency (%)	Outcomes	References
COS7-PCL-ICG, 4T1-PCL-ICG, and B16-PCL-ICG	130-135 nm, spherical	Mouse melanoma (B16F10), breast cancer (4T1), and normal (COS-7)	U87MG intracranial orthotropic glioblastoma mice, 8-hour post-injection mice subjected to laser.	808 nm (1W/cm^2^, 5 minute)	A higher degree of apoptosis was observed in B16 cell membrane camouflaged PCL-ICG NPs compared to 4T1 and normal cell camouflaged PCL-ICG NPs. TThese camouflaging NPs with metastatic tumor cell membrane showed promising BBB penetration and hence can be used for precise therapy of brain tumors.	97
AuNRs/MoS_2_-ICG Nanocomposite	103 nm, the AuNRs were randomly deposited onto the surface of the pegylated MoS_2_ nanosheet	Human umbilical vein endothelial (HUVEC) and cervical cancer (HeLa)	Mice aged 4-5 weeks were injected with HeLa tumor in the armpit, after that 4-hour post-injection of nanocomposite mice subjected to continuous wave laser.	808 nm (0.2 W/cm^2^, 5 minute); 68.8%	The continuous wave single low power laser triggered simultaneous PDT and synergistic PPTT effects of AuNRs/MoS_2_-ICG nanohybrid, which demonstrated a safer treatment approach and was found promising for clinical translation.	104
Au@MgFe_3_O_4_ Nanohybrids	42±7 nm, core (Au) - shell (MgFe_3_O_4_) flowerlike structure	Human hepatoma (HepG2)	Flanks of nude mice with HepG2 cells were injected, and nanohybrids were injected once the solid tumor volume reached 100 mm^3^.	808 nm (0.5 W/cm^2^, 10 minute)	The intra-tumoral administration of the nanohybrid, and laser irradiation, successfully regressed in-vivo tumor growth.	108
AuNR-ICG@rGO-DOX Nanohybrids	Width ~60 nm and length ~90 nm, core-shell structure.	Human colon cancer (HT-29)	Male balb/c mice aged 5 weeks were subcutaneously injected with HT-29, and then nanohybrids were injected once the solid tumor volume reached 100 mm^3^.	808 nm (2 W/cm2, 5-minute)	This multifunctional nanohybrid demonstrated combined catalytic, and chemotherapeutic effects followed by photothermal ablation of tumor growth with minimal side effects on healthy tissues.	12
CeVO_4_/Ag Nanocomposite	127 nm, spherical	Mouse fibroblast (L929) and cervical cancer (HeLa)	6-week-old balb/c mice were injected subcutaneously with H22 cells into the left axilla, followed by nanocomposite injection once the solid tumor volume reached 100 mm^3^.	808 nm (0.7 W/cm^2^, 5 minute); 23.48%	CeVO_4_/Ag showed almost no tumor growth after NIR-triggered PTT/PDT. Histology analysis of major organs showed no noticeable abnormalities or injuries.	109
GNR@Cu_2-x_Se nanohybrid	74.7 nm length, 44.1 nm width, and 8.6 nm thickness with core-shell type heterostructure	Normal liver cells (L-02), and breast cancer (MDA-MB-231)	Female nude mice aged 4 weeks were injected subcutaneously with MDA-MB-231 cells; afterward, the nanocomposites were injected once the solid tumor volume reached 150 mm^3^.	1064 nm (1.0 W/cm^2^, 5 minute); 58-85%	The nanohybrids were found haemocompatible. The temperature rose to 63.6 °C when structures were prepared with CTAB. After the intratumoral administration, these heterostructured nanohybrids stayed longer in the tumor site, resulting in photothermal ablation of the tumor.	106
Iron Oxide Nanoflowers@CuS nanohybrids	120.4±7.3 nm, core-shell assembly	Human prostate adenocarcinoma (PC3)	Immunodeficient nude NMRI female mice (without thymus) aged 9 weeks were injected with PC3 cells in the right and left flanks, then the nanocomposites were injected once the solid tumor volume reached 125 mm^3^.	1064 nm (1.0 W/cm^2^, 10 minutes); 42±6%	Complete tumor regression was achieved for PTT mode compared to MHT (magnetic hyperthermia). This tri-therapeutic strategy enables serial heating cycles leading to lower laser power, reduce in dose of nanoparticle, photoacoustic agents hold promise for clinical translation.	107
Paclitaxel/Palladium pthalocyanine@Hollow Silica polymer Nanohybrid (Pax/Pdpc@HPSN)	Diameter of 21 nm, spherical	HeLa	6-week-old female nude mice with S180 murine sarcoma were injected into the right axilla, and then the nanohybrids were injected once the tumor length reached 70-90 mm.	730 nm (1.9 W/cm^2^)	Tumors are recured if treated alone with PTT treatment. However, it was eradicated when chemotherapy was combined with PTT.	110
Pd@Pt-PEG nanocomposite	Pt shell on Pd nanocube	Murine osteosarcoma (LM8)	6-week-old balb/c female mice were injected with LM8 cells and treated with the nanocomposite once the tumor volume reached 100 mm^3.^	808 nm (1W/cm^2^, 5 minute); 74.5%	The use of PTT alone had no anti-tumor effect. The combination (PTT/PDT) was successful.	111
CuS-^89^Zr-Mesoporous silica nanoshells construct	~ 160 nm in diameter, spherical	4T1	4T1 tumors were injected into the front or hind flanks of balb/c female mice and treated with the nanoconstruct once the tumor volume reached 100 mm^3^.	980 nm (4 W/cm^2^, 10 minutes)	A rapid, complete elimination of the tumor without any side effects or recurrence was observed during the *in-vivo* study*.*	100
Cu/C quantum dots-crosslinked Nanosheets (CuCD NSs)	20-30 nm, spherical	Breast cancer (MCF-7)	Mice with subcutaneous C6 cancer xenografts were used. Hybrids infused in mice were mediated by lysosomal capture after 4 hours of preconditioning with PEG-modified CuCD NS with varying Cu concentrations.	808 nm (2 W/cm^2^, 10 minutes); 41.3%	Upon irradiation, the viability of MCF-7 cells decreased with increasing concentration of PEG-modified CuCD NSs. Laser enhanced the rate of early apoptosis from 5.28 to 80.77%. The PTT effect was enhanced due to laser-triggered cytosolic/nuclear delivery of CuCD NSs.	112
Spiky silver-Iron Oxide Nanohybrid (AgIONPs)	165.3±0.2 nm (hydrodynamic diameter), spherical nanoclusters	Human glioblastoma (U87MG)	10-week-old balb/c mice were subcutaneously injected with U-87 MG cells into the right flank, after that, nanohybrids were injected once the solid tumor volume reached 100-150 mm^3^.	808 nm (0.5-2 W/cm^2^, 5 minutes); 21.4%	The nanohybrids were further made target-specific by folic acid conjugation and a significant reduction in the tumor mass was reported after intravenous injection. Even without irradiation, AgIONPs induced death in cancer cells.	113
Ti_3_C_2_@TiO_2-x_ Nanohybrid	~ 10 nm in diameter, heterostructure	Human embryonic kidney (HEK_293_T), and 4T1	balb/c mice bearing 4T1 tumors treated with nanohybrids and 4-hour post-injection subjected to the laser.	NIR-II: 1064 nm (0.8 W/cm^2^,10 minutes); 35.8% and Ultrasound (US): 1W/cm^2^, 5 minutes	The engineered nanohybrid reported complete tumor ablation owing to their light-triggered PCE and US-stimulated enhanced sonodynamic ROS generation.	114

**Table 5:** Clinical trial study

Type of Study	Nanohybrids	Size	Phase study	Purpose/Condition	Study Status	Clinical trial number	URL
Single dose efficacy study of AuroLase® Therapy in the metastatic lung tumor treatment	Polyethylene glycol coated gold nanoshell nanomaterial Auroshell® used to enhance photothermal therapy through Near Infrared laser irradiation method	120-150 nm	Not applicable	To treat lung cancer, metastatic lung tumor, lung neoplasm,	First posted: 13.08.2012 Completion date: 3.11.2016 (terminated)	NCT01679470	https://www.clinicaltrials.gov/study/NCT01679470?term=NCT01679470&rank=1
		15 nm		Refractory or recurrent tumor of the head and neck	First posted: 20.02.2009 Completion: 09.02.2017 (completed)	NCT00848042	https://www.clinicaltrials.gov/study/NCT00848042?term=NCT00848042&rank=1
				Neoplasm of the prostate tissue	First submitted: 02.2016 Final Submitted:03.03.2021 (not recruiting)	NCT02680535	https://www.clinicaltrials.gov/study/NCT02680535?term=NCT02680535&rank=1
					First submission: 30.01.2020 Final Submission: 18.05.2023 (recruiting)	NCT04240639	https://www.clinicaltrials.gov/study/NCT04240639?term=NCT04240639&rank=1
Doxorubicin Hydrochloride Injection Lipodox® Caelyx® Bioequivalence Study in Patients with Breast and Ovarian Cancer	The free and encapsulated doxorubicin concentrations in plasma were measured using two different, verified liquid chromatography-mass spectrometry analytical techniques.	90 nm	Phase I	Breast and Ovarian cancer	First posted: 28.07.2021 Final posted: 10.03.2022	NCT05273944	https://www.clinicaltrials.gov/study/NCT05273944?term=NCT05273944&rank=1

## Abbreviations

PTT: photothermal therapy
NIR: near infrared
FDA: food and drug administration
LSPR: localized surface plasmon resonance
NP: nanoparticle
PCE: photothermal conversion efficiency
PDT: photodynamic therapy
ICG: indocyanine green
rGO: reduced graphene oxide
DOX: doxorubicin
ROS: reactive oxygen species
PEG: polyethylene glycol
RGD: arginine-glycine-aspartic acid
POD: peroxidase
EPR: enhanced permeability and retention
SWCNTs: single-walled carbon nanotubes
MSNs: mesoporous silica nanoparticles
CNTs: carbon nanotubes
MTT: 3-[4,5-dimethylthiazol-2-yl]-2,5 diphenyl tetrazolium bromide
MIC: minimum inhibitory concentration
CTCAE: common terminology criteria for adverse events
